# Cluster analysis of patient characteristics, treatment modalities, renal impairments, and inflammatory markers in diabetes mellitus

**DOI:** 10.1038/s41598-024-56451-1

**Published:** 2024-03-12

**Authors:** Milena Cojic, Aleksandra Klisic, Amina Sahmanovic, Nemanja Petrovic, Gordana Kocic

**Affiliations:** 1 University of Montenegro-Faculty of Medicine, Podgorica, Montenegro; 2 Primary Health Care Center, Podgorica, Montenegro; 3 Department of Medical Biochemistry, School of Medicine, University of Nis, Niš, Serbia

**Keywords:** Type 2 diabetes mellitus, Inflammation, Neutrophil-to-lymphocyte ratio, Platelet-to lymphocyte ratio, Clustering, glomerular filtration rate, Biomarkers, Endocrinology

## Abstract

Type 2 diabetes mellitus (T2DM) is caused by an interplay of various factors where chronic hyperglycemia and inflammation have central role in its onset and progression. Identifying patient groups with increased inflammation in order to provide more personalized approach has become crucial. We hypothesized that grouping patients into clusters according to their clinical characteristics could identify distinct unique profiles that were previously invisible to the clinical eye. A cross-sectional record-based study was performed at the Primary Health Care Center Podgorica, Montenegro, on 424 T2DM patients aged between 30 and 85. Using hierarchical clustering patients were grouped into four distinct clusters based on 12 clinical variables, including glycemic and other relevant metabolic indicators. Inflammation was assessed through neutrophil-to-lymphocyte (NLR) and platelet to lymphocyte ratio (PLR). Cluster 3 which featured the oldest patients with the longest T2DM duration, highest hypertension rate, poor glycemic control and significant GFR impairment had the highest levels of inflammatory markers. Cluster 4 which featured the youngest patients, with the best glycemic control, the highest GFR had the lowest prevalence of coronary disease, but not the lowest levels of inflammatory markers. Identifying these clusters offers physicians opportunity for more personalized T2DM management, potentially mitigating its associated complications.

## Introduction

Diabetes mellitus is a chronic metabolic disorder that plays a substantial role in escalating global health challenges. Type 2 diabetes mellitus (T2DM), which accounts for 90% of all diabetes cases, eventually leads to complications that significantly impair the quality of life, resulting in premature disability and death^1,2^. Complications can be classified as microvascular which primarily target the retina, kidneys, and nerves, leading to conditions like retinopathy, nephropathy, and neuropathy and on the other hand, macrovascular which affect larger blood vessels, predisposing individuals to cardiovascular diseases (CVD) such as coronary artery disease, stroke, and peripheral arterial disease^3^. The complex relationship between T2DM and atherosclerotic CVD is well-established, signifying 2–4 times augmented risk for cardiovascular death among diabetic patients^4^. Therefore, the CVD is of a great concern in the T2DM progression with recent evidence supporting strong interconnection between microvascular and macrovascular disorders. in T2DM, pointing out the potential of microvascular diseases in promoting athero-sclerosis through additional indirect mechanisms^5–7^.

Chronic hyperglycemia remains the most critical factor for the onset and progression of diabetes related complications by inducing various metabolic and biochemical imbalances. On the other hand, more and more research is being conducted highlighting inflammation as the central pathological mechanism underlying these complications^8^. Supporting this, research evidence show that good glycemic control can attenuate the risk for T2DM and its complications to some extent, but comprehensive care for individuals with T2DM often necessitates the management of other risk factors such as hypertension and dyslipidemia, as well as lifestyle modifications^9^.

New classes of drugs that have been developed act on different systems and they not only reduce hyperglycemia, but have beneficial effects on other cardiovascular risk factors like reducing blood pressure, reducing progression of renal impairments and promoting weight loss^10^. They have also shown anti-inflammatory effects^11^. Therefore, the choice of a pharmacological agent should be based on a holistic understanding of the patient's clinical profile, including the potential role of inflammation.

Concerning the management of this multifactorial chronic disease, a special attention should be paid on the older adult population given the fact that they face with frequent occurrence of comorbidities^12^. This imposes the need for an individual approach in the adults with T2DM. Glycemic control targets also differ between the different population groups. The less stringent target HbA1c has been recommended for older T2DM patients with multiple comorbidities and long duration of diabetes^13^. Therefore, the need to divide T2DM patients into phenotypes, in order to bring closer the concept of complexity of T2DM is of utmost importance.

Within this context, grouping patients into clusters according to their clinical characteristics appears as a very useful tool in identifying patterns and nuances that were previously invisible to the clinical eye, which was also the aim of the present study. This could help clinicians to tailor interventions ensuring that each patient receives care in accordance with their unique profile, potentially minimizing the risk of complications and comorbidities.

## Results

The study included 424 patients, 208 males and 216 females. The average age of the population was 66.19 ± 11.14 years, ranging from 26 to 91 years. Participants were predominately non-smokers with average duration of the disease less than 10 years (8.67 ± 4.93 years). About 90% had hypertension, with an average SBP of 134 mmHg and DBP of 82.11 mmHg.

Table 1 presents the basic demographic and clinical features of the study population.

**Table 1:** Demographic and clinical characteristics data of the study population.

	Mean ± SD/Count	Min–Max/%
Age (years)	66.19 ± 11.14	26–91
Age of onset (years)	57.52 ± 10.63	21–87
BMI (kg/m^2^)	28.34 ± 4.62	17.40–43.27
Gender		
Male	208	49.1
Female	216	50.9
T2DM duration (years)	8.67 ± 4.93	1.0–15.0
Smoking status		
Non-smoker	245	57.8
Smoker	93	21.9
Ex-smoker	74	17.5
SBP (mmHg)	134 ± 14.28	90–190
DBP (mmHg)	82.11 ± 9.05	45–120
Hypertension	382	90.1
Antihypertensives	377	88.9
Angiotensin-Converting Enzyme inhibitors	302	71.2
Beta blockers	238	56.1
Calcium antagonists	101	23.8
Diuretics	242	57.1
Angiotensin II Receptor Blockers	33	7.8
Hyperlipidemia	349	82.3
Statins	251	59.2
Neuropathy	109	25.7
Retinopathy	48	11.3
Nephropathy	73	17.2
Coronary artery disease	144	34.0
Myocardial infraction	50	11.8
Grafts	64	15.1
Stroke	20	4.7
Peripheral artery disease	18	4.2
Heart failure	11	2.6
Antiplatelet agents	201	47.4
Diabetes treatments		
DNT	278	65.5
DIT	33	7.8
DNIT	113	26.7
GFR category		
G1	205	48.3
G2	148	34.9
G3a	42	9.9
G3b	17	4.0
G4	11	2.6
G5	1	0.2

BMI, body mass index; SBP, systolic blood pressure; DBP, diastolic blood pressure; DNT, Diet + non-insulin antidiabetic therapy; DIT, Diet + insulin therapy; DNIT, non-insulin antidiabetic agents and insulin; GFR, glomerular filtration rate.

Coronary disease was the most prevalent manifestation of CVD. Hyperlipidemia was spotted in 82.3% of patients, but only 60% of them were using hypolipidemic drugs (i.e. statins). Most patients were on non-insulin medications, and over a third (34.5%) were receiving insulin treatment.

The average serum glucose level was 7.91 ± 2.95 mmol/L and HbA1c 7.28 ± 1.66% suggesting that many patients had elevated blood sugar levels. Most of the patients had decreased levels of GFR and increased levels of ESR (Table 2).

**Table 2:** Laboratory characteristics of the study population.

	Mean ± SD/Count	Min–Max
Fasting glycaemia (mmol/L)	7.91 ± 2.95	3.1–22.1
HbA1c (%)	7.28 ± 1.66	4.51–15
Urea (mmol/L)	7.25 ± 3.69	1.90–28.80
Creatinine (μmol/L)	83.67 ± 43.88	6.5–572.0
GFR (mL/min per 1.73m^2^)	82.42 ± 22.99	7–159
TC (mmol/L)	5.16 ± 1.26	2.53–10.60
HDL (mmol/L)	1.26 ± 0.35	0.55–2.95
LDL (mmol/L)	2.94 ± 1.02	0.72–6.93
TRG (mmol/L)	2.25 ± 1.8	0.4–22.7
AST (IU/L)	25.23 ± 14.18	9–157
ALT (IU/L)	28.49 ± 23.74	6–243
ESR (mm/h)	16.79 ± 15.03	2–84
WBC (10^9^/L)	7.62 ± 2.06	2.95–16
Neutrophils (10^9^/L)	4.14 ± 1.53	1.38–9.2
Lymphocytes (10^9^/L)	2.59 ± 0.9	0.82–7.06
NLR	1.79 ± 1.01	0.51–7.34
PLT (10^9^/L)	261.72 ± 72.92	69.00–261.73
PLR	112.99 ± 56.34	24.64–529.27

HbA1c, hemoglobin A1c; GFR, glomerular filtration rate; TC, total cholesterol; HDL, high-density lipoprotein; LDL, low-density lipoprotein; TRG, triglycerides; AST, aspartate transaminase; ALT, alanine transaminase; ESR, erythrocyte sedimentation rate; WBC, white blood count; NLR, neutrophil to lymphocyte ratio; PLT, platelets; PLR, platelet to lymphocyte ratio.

Clustering was performed based on previously mentioned variables. The first cluster included 48 patients, the second 211 patients, the third 73 and the fourth 92 patients. Clusters differed significantly in relation to age (p < 0.001), T2DM duration (p < 0.001), HbA1c values (p < 0.001), urea (p < 0.001), creatinine (p < 0.001), GFR (p < 0.001), TC (p = 0.025), LDL (p < 0.001), frequency of renal impairment (p < 0.001), hypertension (p < 0.001) and smoking status (p = 0.026). The frequency of diabetes medication use like sulfonylurea (p < 0.001), GLP-1r (p = 0.007), SGLT2i (p < 0.001), DDP4 (p < 0.01), and insulin (p < 0.001) also showed significant differences between clusters (Table 3).

**Table 3:** Clinical characteristics of clusters.

Cluster	Cluster 1		Cluster 2		Cluster 3		Cluster 4		p^1^
	n = 48		n = 211		n = 73		n = 92		
Age (years)	66.77 ± 7.56		67.23 ± 10.00		71.23 ± 9.04^b^		59.52 ± 13.56^a,b^		< 0.001
Fasting glycaemia (mmol/L)	8.69 ± 2.85		7.84 ± 2.8^a^		8.28 ± 3.79		7.36 ± 2.43^a^		0.040^2^
BMI (kg/m^2^)	29.24 ± 3.59		28.22 ± 4.7		28.97 ± 4.56		27.66 ± 4.91		0.151
T2DM duration (years)	7.28 ± 4.94		9.08 ± 4.84^a^		10.11 ± 4.5^a^		7.3 ± 5.04^b,c^		< 0.001
HbA1c (%)	8.17 ± 1.88		7.23 ± 1.57^a^		7.35 ± 1.79^a^		6.85 ± 1.47^a,b,c^		< 0.001
Urea (mmol/L)	7.2 ± 3.12		6.79 ± 2.22		11.08 ± 6.03^a,b^		5.29 ± 1.41^b,c^		< 0.001
Creatinine (μmol/L)	79.42 ± 26.38		77.79 ± 18.69		130.44 ± 81.64^a,b^		62.25 ± 14.25		< 0.001
GFR (mL/min per 1.73m^2^)	83.46 ± 22.01		83.01 ± 15.77		57.32 ± 29.56^a,b^		100.45 ± 10.28^a,b,c^		< 0.001
TC (mmol/L)	5.46 ± 0.93		5.21 ± 1.36		4.79 ± 1.19^b^		5.2 ± 1.20^c^		0.025
HDL (mmol/L)	1.24 ± 0.27		1.24 ± 0.33		1.29 ± 0.40		1.31 ± 0.38		0.362
LDL (mmol/L)	3.27 ± 0.84		3 ± 1.09^a^		2.52 ± 0.91^a,b^		2.98 ± 0.92^a,c^		< 0.001
TRG (mmol/L)	2.17 ± 1.13		2.34 ± 1.67^a^		2.12 ± 1.23		2.2 ± 2.58^a,b^		0.800
Metformin	41	85.4	175	82.9	54	74.0	67	73.6	0.121
Sulfonylurea	47	97.9	15	7.1	4	5.5	0	0.0	< 0.001
GLP-1r	0	0.0	15	7.1	6	8,2	1	1.1	0.007
SGLT2i	11	22.9	106	50.2	12	16.4	16	17.6	< 0.001
DDP4i	6	12.5	36	17.1	26	35.6	4	4.4	< 0.001
Insulin	6	12.5	80	37.9	31	42.5	25	27.5	0.001
GFR groups									
G1	24	50.0	73	34.6	16	21.9	92	100.0	< 0.001
G2	15	31.3	127	60.2	6	8.2	0	0.0	
G3a, G3b, G4, G5	9	18.8	11	5.2	51	69.9	0	0.0	
Smoking status									
Non-smoker	21	46.7	118	57.3	50	70.4	56	62.2	0.026
Smoker	17	37.8	52	25.2	11	15.5	13	14.4	
Ex-smoker	7	15.6	36	17.5	10	14.1	21	23.3	
Hypertension	44	91.7	195	82.4	71	97.3	72	78.3	< 0.001
CVD	15	31.3	88	41.7	36	49.3	18	19.6^b,c^	< 0.001

BMI, body Mass Index; T2DM, type 2 diabetes mellitus; HbA1c, hemoglobin A1c; GFR, glomerular filtration rate; TC, total cholesterol; HDL, high-density lipoprotein; LDL, low-density lipoprotein; TRG, triglycerides; GLP-1r, glucagon-likepeptide-1 receptor agonists; SGLT2i, sodium-glucose cotransporter-2 inhibitors; DPP4i, dipeptidyl peptidase-4 inhibitors.

^1^ANOVA, ^2^Kruskal–Wallis’s test, ^3^Chi-squared test, ^a^vs Cluster 1 p < 0.05, ^b^vs Cluster 2 p < 0.05, ^c^vs Cluster 3 p < 0.05.

The Cluster 3 included the oldest patients (p < 0.001) with the longest duration of T2DM (p < 0.001) and the highest percentage of hypertension (p < 0.001). Patients from cluster 3 had the highest mean level of urea (p < 0.001), creatinine (p < 0.001), and the lowest GFR value (p < 0.001). This cluster had the highest percentage of patients diagnosed with significant GFR impairment (G3–G5) (p < 0.001) and the highest percentage of patients using DDP4i (p < 0.001) and insulin (p < 0.001). The Cluster 4 included the youngest patients, with the shortest disease duration, the lowest levels of fasting glycaemia (p = 0.040) and HbA1c (p < 0,001). Patients in this cluster also had the lowest mean levels of urea and creatinine, and the highest GFR level (Table 3). The prevalence of CVD was significantly different among clusters (p < 0.001). CVD were significantly more prevalent in Cluster 2 and Cluster 3 compared to Cluser 4 (p < 0.001 for both).

When comparing inflammatory markers, as shown in Table 4 there is a statistically significant difference between clusters regarding levels of NLR (p < 0.001), PLR (p = 0.001), ALT (p = 0.039), neutrophil count (p = 0.024), and lymphocyte count (p = 0.010). The Cluster 3 had significantly higher levels of NLR, PLR, neutrophil count and lower levels of lymphocyte count compared to the Cluster 1 (p < 0.001, p = 0.002, p = 0.025, p = 0.001), the Cluster 2 (p < 0.001, p = 0.008, p = 0.022, p = 0,042), and the Cluster 4 (p < 0.001, p = 0.010, p = 0.007, p = 0,016). The categories of NLR differed significantly between clusters (p < 0.001) meaning that the Cluster 3 had the highest number of patients with NLR levels ≥ 2. The prevalence of neuropathies (p = 0.009), nephropathies (p < 0.001), and coronary artery diseases (p < 0.001) were the highest in the Cluster 3.

**Table 4:** Clinical characteristics and inflammation markers in relation to the cluster analysis.

Characteristic	Cluster 1		Cluster 2		Cluster 3		Cluster 4		p^1^
Gender									
Male	23	47.9	109	51.7	30	41.1	46	50.0	0.477
Female	25	52.1	102	48.3	43	58.9	46	50.0	
NLR	1.49 ± 0.76		1.72 ± 0.80^a^		2.31 ± 1.41^a,b^		1.68 ± 1.05^c^		< 0.001^2^
PLR	98.34 ± 34.52		109.86 ± 46.17		135.44 ± 83.38^a,b^		109.99 ± 56.21^c^		0.001^2^
PLT (10^9^/L)	256.25 ± 51.69		260 ± 70.99		270.48 ± 83.15		261.6 ± 78.41		0.814^2^
AST (IU/L)	27.34 ± 23.46		25.66 ± 14.93		24.64 ± 10.3		23.55 ± 6.13		0.794^2^
ALT (IU/L)	34.02 ± 37.00		28.99 ± 24.80		23.26 ± 14.82		28.5 ± 16.41		0.039^2^
ESR (mm/h)	17.26 ± 14.65		16.27 ± 14.24		20.52 ± 16.52		14.63 ± 16.1		0.177^2^
WBC (10^9^/L)	7.74 ± 2.17		7.58 ± 2.10		7.82 ± 2.01		7.5 ± 1.95		0.718^2^
Neutrophils (10^9^/L)	3.93 ± 1.59		4.12 ± 1.51		4.6 ± 1.59^a,b^		3.95 ± 1.47^c^		0.024^2^
Lymphocytes (10^9^/L)	2.83 ± 0.89		2.59 ± 0.89^a^		2.29 ± 0.78^a,b^		2.69 ± 0.97^c^		0.010^2^
SBP (mmHg)	134.39 ± 14.58		134.51 ± 13.76		136.31 ± 14.89		131.8 ± 14.7		0.239^3^
DBP (mmHg)	82.61 ± 9.08		82.22 ± 8.67		82 ± 9.63		81.69 ± 9.56		0.947^3^
Antihypertensives	45	97.8	188	90.4	71	98.6^b^	73	80.2^a,b,c^	< 0.001
Angiotensin-Converting Enzyme inhibitors	39	83.0	144	69.9	59	81.9	60	66.7^c^	0.044
Beta blockers	27	57.4	120	59.4	50	71.4	41	47.1^c^	0.023
Calcium antagonists	13	28.3	49	24.7	22	32.4	17	20.2	0.369
Diuretics	29	64.4	117	57.9	54	77.1^b^	42	49.4^c^	0.004
Angiotensin II Receptor Blockers	2	4.4	19	9.7	5	7.6	7	8.5	0.709
Antiplatelet agents	18	37.5	111	53.6	45	62.5^a^	27	30.0^b,c^	< 0.001
Diabetes treatment									
DNT	41	85.4	130	61.6	41	56.2	66	71.7^b,c^	0.001
DIT	0	0.0	14	6.6	7	9.6	12	13.0	
DNIT	7	14.6	67	31.8	25	34.2	14	15.2	
Grafts	6	12.5	39	18.5	13	17.8	6	6.5^b,c^	0.049
Statins	25	53.2	127	61.1	50	69.4	49	53.3	0.144
Hyperlipidemia	38	79.2	178	84.4	64	87.7	69	75.0	0.133
Neuropathy	6	12.5	66	31.3^a^	21	28.8	16	17.4^b^	0.009
Retinopathy	3	6.3	19	9.0	13	17.8	13	14.1	0.105
Nephropathy	9	18.8	14	6.6^a^	50	68.5^a,b^	0	0.0^a,b,c^	< 0.001
Coronary artery disease	14	29.2	80	37.9	34	46.6	16	17.4^b,c^	< 0.001
Myocardial infraction	7	14.6	23	10.9	13	17.8	7	7.6	0.203
Stroke	2	4.2	11	5.2	6	8.2	1	1.1	0.184
Peripheral artery disease	2	4.2	10	4.7	4	5.5	2	2.2	0.712
Heart failure	3	6.3	4	1.9	2	2.7	2	2.2	0.389
NLR									
0.1–0.9	12	25.0	23	10.9	5	6.8^a,b^	19	20.7^c^	< 0.001
1.0–1.9	26	54.2	131	62.1	36	49.3	54	58.7	
2.0–2.9	9	18.8	46	21.8	18	24.7	12	13.0	
3.0–7.0	1	2.1	11	5.2	14	19.2	7	7.6	

NLR, neutrophil to lymphocyte ratio; PLR, platelet to lymphocyte ratio; PLT, platelets; AST, aspartate transaminase; ALT, alanine transaminase; ESR, erythrocyte sedimentation rate; WBC, white blood count; SBP, systolic blood pressure; DBP, diastolic blood pressure; DNT, Diet + non-insulin antidiabetic therapy; DIT, Diet + insulin therapy; DNIT, non-insulin antidiabetic agents and insulin.

^1^Chi-squared test, ^2^Kruskal–Wallis’s test, ^3^ANOVA, ^a^vs Cluster 1 p < 0.05, ^b^vs Cluster 2 p < 0.05, ^c^vs Cluster 3 p < 0.05;

In multivarate regression analysis (Backward Wald method) it is established that CVD is significantly associated with gender (p = 0.016), T2DM duration (p = 0.041) in Cluster 1, with age of onset (p = 0.004) and HbA1c (p = 0.039) in Cluster 2, with age of T2DM onset (p = 0.030), T2DM duration (p = 0.003), HbA1c (p = 0.023), and NLR (p = 0.029) in Cluster 3, and with age of onset (p = 0.029) in Cluster 4 (Table 5).

**Table 5:** Association of CVD and age of onset, gender, T2DM duration, fasting glycaemia, HbA1c, NLR in four clusters.

		B	S.E	OR	95% C.I.for OR		p
					Lower	Upper	
Cluster 1	Age of T2DM onset	0.105	0.059	1.111	0.990	1.246	0.074
	Gender	− 2.195	0.911	0.111	0.019	0.664	0.016
	T2DM duration	0.198	0.097	1.219	1.008	1.474	0.041
	Fasting glycaemia	− 0.286	0.152	0.751	0.558	1.011	0.059
	Constant	− 2.912	3.492	0.054			0.404
Cluster 2	Age of T2DM onset	0.049	0.017	1.050	1.016	1.085	0.004
	T2DM duration	0.056	0.032	1.057	0.993	1.126	0.083
	Fasting glycaemia	− 0.127	0.070	0.881	0.768	1.010	0.069
	HbA1c	0.266	0.129	1.305	1.014	1.681	0.039
	Constant	− 4.616	1.345	0.010			0.001
Cluster 3	Age of T2DM onset	0.079	0.036	1.082	1.008	1.161	0.030
	T2DM duration	0.250	0.086	1.285	1.086	1.520	0.003
	HbA1c	− 0.513	0.225	0.598	0.385	0.930	0.023
	NLR	0.665	0.305	1.945	1.070	3.535	0.029
	Constant	− 5.189	3.080	0.006			0.092
Cluster 4	Age of T2DM onset	0.056	0.026	1.058	1.006	1.113	0.029
	Constant	− 4.562	1.504	0.010			0.002

B, regression coefficient; S.E., standard error; OR, odds ratio; 95% C.I., confidence interval.

## Discussion

When treating patients with T2DM, one of the main challenges physicians face is understanding the complexity of individual patient profiles. This complexity arises not only from the multidimensional nature of T2DM itself, but also from the multitude of associated comorbidities and underlying pathophysiological processes^14,15^. It implies that course of the disease can be highly variable. While some patients with T2DM face rapid deterioration, others maintain stable for extended period of time. This makes long-term planning challenging^16^.

Recognizing the important role of inflammation in the T2DM pathophysiology and its associated comorbidities has highlighted the need to identify groups of patients prone to increased inflammation as well as to identify risk factors associated with increased inflammatory responses^17^. In this way clinicians can more effectively adjust therapeutic approaches and provide better control of the disease that extends beyond glucose control.

According to the authors’ best knowledge, the studies that applied clustering method to group the patients with T2DM targeting inflammation, comorbidities and therapy regimens are scarce. We found only one study that used clustering to pair inflammatory and clinical parameters in patients with T2DM. However, it was conducted on a considerably smaller sample size than our study^14^.

We identified four distinct profiles of patients with T2DM based on their clinical and demographic characteristics. Each cluster had its unique characteristics and differed in terms of age, disease duration, associated conditions, and biochemical profiles. The Cluster 3 featured the oldest patients with the longest duration of T2DM, who also had the lowest levels of GFR and exhibited poor glycemic control. Patients from this cluster had the highest level of NLR and PLR which means that the Cluster 3 had the most pronounced subclinical inflammation since the correlation between increased NLR and PLR values and inflammation in T2DM is well established in the literature^18^. These findings are in alignment with other studies suggesting that inflammation might be associated with a more advanced or prolonged stage of T2DM, poor glycemic control and low GFR^19–22^.

T2DM is considered to be age-related disease. It is characterized by chronic activation of the innate immune system which can be increased by over-nutrition and aging process^23^. Over-nutrition in addition to genetic predisposition and lack of physical activity leads to obesity. Particularly in cases of central adiposity, this can trigger adipose tissue dysfunction, prompting macrophage infiltration and a subsequent surge in inflammatory cytokine release^24^. Chronically elevated inflammatory biomarkers promote insulin resistance and hyperglycemia. Furthermore, chronic hyperglycemia sustains persistent inflammation creating a cycle where inflammation exacerbates glucose metabolic disturbances, further aggravating the body's metabolic equilibrium^24^. This can explain why patients with higher levels of HbA1c like in the Cluster 3 exhibit the higher level of inflammation, as determined by higher NLR, PLR and neutrophil count.

Chronic hyperglycemia and inflammation have detrimental effects on various organs including kidneys^25^. These effects manifest as changes in the microvasculature, particularly in the thickening of the capillary basement membrane impacting arterioles in the glomeruli, retina, myocardium, skin, and muscle. Such alterations in the glomeruli play a crucial role in the onset and progression of diabetic nephropathy^6^. In a recent study, it was found that an increased NLR and PLR were not only significantly correlated with diabetic nephropathy but were also proposed as predictors and prognostic risk markers of diabetic nephropathy^26^. Our findings align with this, highlighting the interrelationship between kidney function and inflammatory responses. Specifically, once kidneys are damaged, they can further exacerbate inflammatory responses in the body^27^. This interplay is reflected in the Cluster 4, where good kidney function corresponds well with moderate inflammation markers, potentially suggesting a protective mechanism against intense inflammation. Furthermore, the Cluster 3 had the highest percentage of patients diagnosed with diabetic nephropathy and coronary disease. Previous studies also showed that higher NLR level was associated with an increased prevalence of CVD and diabetic nephropathy pointing out the important role that inflammation plays in development of such complications^28^. The coexistence within a single cluster highlights their interconnected nature^6,7^.

Patients in Cluster 4, who are characterized with the best clinical performances, have higher mean level of inflammation (as indicated by NLR and PLT markers) than patients in Cluster 1 (who have lower renal function and more CVD and worse metabolic indicators) but this difference did not reach statistical significance. Another feature of the clusters is the fact that patients in Cluster 4 have significantly lower level of inflammation (as indicated by NLR and PLT markers) than patients in Cluster 3, who are the worst with respect to the presence of CV comorbidities, and are also the oldest ones. One of the possible reasons for such discrepancies includes the wide range of age of studied diabetic patients that could have influenced the characteristics of clusters, in addition to differences in medications use. Furthermore, there are complex relationships between age, gender, postmenopausal status, T2DM duration, body shape, BMI categories, HbA1c, and inflammatory marker values as observed in previous studies^29,30^.

Although the Cluster 3 had the highest levels of inflammatory markers, we observed paradoxically low levels of total cholesterol and LDL. Considering the high prevalence of coronary artery disease in the Cluster 3, it might be plausible that these patients have been treated aggressively with lipid-lowering therapies in the past or might still be under such treatment.

Notably, the Cluster 3 also demonstrated a pronounced percentage of retinopathy cases, although this association did not reach statistical significance. These results are in line with a study conducted by Ciray et al.^31^ that found no independent association between NLR and diabetic retinopathy. While some research has suggested NLR as a potential diagnostic biomarker for diabetic retinopathy, the association remains debated^28^. The highest percentage of patients with diabetic neuropathy was in the Cluster 2 which also had the pronounced levels of NLR and PLR but significantly lower than in the Cluster 3. This could be explained by multifactorial nature of the retinopathy and neuropathy where inflammation is just one aspect of a broader pathophysiological picture^32^.

Furthermore, it’s worth noting that Cluster 4 which included the youngest patients with the lowest levels of fasting glycaemia and HbA1c, along with the highest GFR and relatively short disease duration presented with surprisingly higher inflammation markers compared to Cluster 1. Patients from the Cluster 1 also showed some unfavorable characteristics like patients from Cluster 2 and 3 including older patients with high percentage of hypertension and decreased GFR who had the worst glycemic and lipid control. Yet, despite these seemingly adverse factors, this cluster surprisingly exhibited the lowest levels of inflammatory markers. Medication regimen could be a contributing factor to these observed levels of inflammation in the Cluster 1. Namely, these group of patients had the highest percentage of patients on oral therapy, with Metformin being the most commonly used. Even though there was not a statistically significant difference between the clusters regarding Metformin use, we believe its presence played a pivotal role in reducing inflammation levels as suggested in different studies which showed that Metformin has potent anti-inflammatory effect through inhibiting secretion of pro-inflammatory cytokines from activated macrophages^33,34^. In accordance with this, study by Mohammed et al.^35^ revealed a dose-dependent effect of Metformin on the reduction of NLR in T2DM patients. Furthermore, Cluster 1 had the highest percentage of patients using sulfonylureas, which also appear to have some anti-inflammatory effect but less potent than metformin^36^. Despite the documented anti-inflammatory properties of insulin evidenced by both in vitro and animal studies—such as modulation of molecular pathways, reduction of pro-inflammatory cytokine expression, and augmentation of anti-inflammatory mediators—this cluster had the lowest percentage of patients using insulin^37^.

The Cluster 1 had the highest percentage of patients using Angiotensin-Converting Enzyme Inhibitors. These drugs, while primarily recognized for their antihypertensive effects, also exhibit anti-inflammatory, antiproliferative, and antioxidant properties through their action on angiotensin II receptors^38^. This could have further contributed to the reduced inflammation levels observed in this cluster. Although with the lowest levels of inflammatory markers, patients from the Cluster 1 still had the higher percent of patients with diabetic complications, especially coronary artery disease compared to Cluster 4. It is possible that current snapshot of inflammatory markers might not provide a comprehensive history and inflammation may have decreased over time, perhaps due to medication or lifestyle modifications still resulting in coronary artery disease from previously elevated inflammation. However, it is crucial to emphasize that the Cluster 3 showed the highest level of inflammation and had the most pronounced incidence of coronary artery disease. This correlates with findings from prior research indicating that an elevated NLR is closely associated with the progression of coronary atherosclerosis. Increased ratios typically align with a deteriorating cardiovascular risk profile and increased complexity and severity of coronary artery disease confirming the established relationship between inflammation and cardiovascular complications in T2DM patients^39^.

We expected a higher percentage of patients to be using medications with proven cardiovascular and renal benefits (SGLT2i, GLP-1r) in Cluster 3, as it had the highest percentage of patients with renal impairment and coronary heart disease^40^. These drug classes have shown superiority in terms of cardiovascular and renal outcomes compared to DPP4i in patients with T2DM, as demonstrated in a meta-analysis that included 23 cardiovascular outcome trials^41^. However, in addition to Metformin, patients from this cluster more commonly used DPP4i. Other studies have yielded similar results, indicating that despite the proven benefits of SGLT2i and GLP-1r, physicians predominantly continue to prescribe DPP4i. This trend can be explained by clinical inertia^42^.

Our study had some limitations. The first limitation is a cross-sectional design of the study since it allows us to observe association between variables, but it limits us when making casual conclusions. Also, the wide range of age of diabetic patients included in the study could have influenced the characteristics of clusters. Another limitation derived from record based data, because there might be inaccuracies or missing information from medical records. For instance, there could be potential underreporting or misclassification of some clinical conditions based on the ICD-10 codes. While use of prescribed medication was recorded we did not provide data about dietary habits and consumption of over the counter drugs which could both influence inflammation levels.

In conclusion, it is worth to note that inflammation is one of the key contributors to disease T2DM pathophysiology and it is associated with variables like age, disease duration, glycemic control, kidney function and medication regimens. Still, it is important to emphasize that inflammation is not the only factor contributing to the development and progression of T2DM and its complications. Other factors like genetic predisposition, comorbidities, lifestyle choices, changes in metabolic control over the time all play significant role in disease progression. This also emphasizes the need to personalize approach in managing T2DM. In that sense, the identification of these distinct clusters provides invaluable insights. Beyond glycemic control, an integrated approach considering inflammation, vascular health, renal function, and other comorbidities is crucial. Further studies are needed to validate and expand these observations.

## Materials and methods

This record based-cross-sectional study was carried out in Primary Health Care Center Podgorica, Montenegro. It included patients from 30 to 85 years who were previously diagnosed with T2DM (International Classification of Diseases 10 [ICD-10] codes E11 and E14) before January 1 2022. Eligible participants were randomly chosen from patients who underwent their regular laboratory assessments between February 1 and April 30, 2022. For each patient we collected the following data that have been recorded until the end of 30.04.2022: age; sex; disease duration; smoking status, the presence of: retinopathy (ICD-10codes E11.3 and H36.0); neuropathy (ICD-10 code G63.2); nephropathy (ICD code E11.21); coronary artery disease (ICD-10 codes I20, I21, I22, I23 and I24); stroke (ICD-10 codes I63, I64, G45 and G46), peripheral arterial vessel diseases (ICD-10 code I73.9) and chronic heart failure (ICD-10 code I50). Additionally, we collected information on the medications that patients were taking, including antihypertensives, hypolipidemic drugs, antiplatelet agents, and diabetes treatments. The diabetes treatments were categorized as: Diet combined with non-insulin antidiabetic therapy (DNT); Diet with a combination of non-insulin antidiabetic agents and insulin (DNIT); and Diet alongside insulin therapy (DIT). Results of laboratory tests were also collected including: white blood count (WBC), platelets (PLT), erythrocyte sedimentation rate (ESR), urea, creatinine, total cholesterol (TC), triglycerides (TRG), low-density lipoprotein (LDL), high-density lipoprotein (HDL), alanine transaminase (ALT), aspartate transaminase (AST), fasting glucose, and glycated hemoglobin (HbA1c). Based on the serum creatinine level we calculated the glomerular filtration rate (GFR) using the Chronic Kidney Disease Epidemiology Collaboration (CKD-EPI) creatinine equation^43^. Subsequently, according to the Kidney Disease: Improving Global Outcomes (KDIGO) 2012 guidelines and GFR readings, patients were stratified into six groups: G1—those with a GFR ≥ 90 mL/min per 1.73m^2^ (normal or high); G2—GFR ranging from 60 to 89 mL/min per 1.73m^2^ (mildly decreased); G3a—GFR between 45 and 59 mL/min per 1.73m^2^ (mildly to moderately decreased); G3b—GFR from 30 to 44 mL/min per 1.73m^2^ (moderately to severely decreased); G4—GFR between 15 and 29 mL/min per 1.73m^2^ (severely decreased); and G5—those with a GFR < 15 mL/min per 1.73m^2^ (kidney failure)^44^. Inflammation markers were determined through the Neutrophil to Lymphocyte Ratio (NLR) and Platelet to Lymphocyte Ratio (PLR) which were estimated by taking the ratio of absolute neutrophil and platelet counts to absolute lymphocyte counts, respectively^18,21,45^.

To further evaluate cardiovascular risk factors, we considered the most recently reported body mass index (BMI) as well as mean values of systolic (SBP) and diastolic blood pressure (DBP) over the previous 12 months.

### Statistical analysis

Data are presented as mean ± standard deviation, frequencies, and percentages. We analyzed data in R using two-step clustering method similar to Ahlqvist and colleagues^46^.

**Figure 1:**
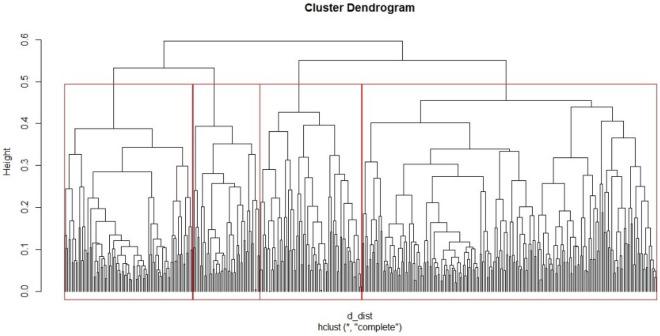
Hierarchical clustering of the diabetes patients.

In the first step, the optimal number of clusters was determined to be 4 by using silhouette analysis (using the pam function) on a series ranging from 2 to 8 clusters. In the second step, hierarchical clustering with Gower distances (accommodate continuous, categorical, and binary variables) was performed to determine different profiles of diabetes patients. The dendrogram (Fig. 1) visualizes the results of patient clustering based on the following variables: age, BMI, T2DM duration, smoking status, hypertension, metformin, sulfonylurea, glucagon-likepeptide-1 receptor agonists (GLP-1r), sodium-glucose cotransporter-2 inhibitors (SGLT2i), dipeptidyl peptidase-4 inhibitor (DPP4i), fasting glycaemia, Hba1c, urea, creatinine, GFR, GFR category, TC, HDL, LDL, TRG. Analysis of variance (ANOVA) and Kruskal–Wallis test were used to evaluate potential differences across different clusters. As a post-hoc analysis we used Tukey or Mann–Whitney test, as appropiate.

The Chi-squared test was used to compare categorical variables across different clusters. A p < 0.05 was considered significant. Multivariate regression analysis (Backward Wald method) was used to estimate the association between CVD and demographic and clinical characteristics in the study population. All statistical analysis was performed using R version 4.1.3 software^47^.

### Institutional review board statement

The study was conducted in accordance with the Declaration of Helsinki, and approved by the Ethics Committee of Primary Health Care Center, Podgorica, Montenegro (ID number 05/17–5946/1, 28.06.2022).

### Informed consent

Informed consent was obtained from all subjects involved in the study. Written informed consent has been obtained from the patient(s) to publish this paper.

## Data Availability

The data will be available upon reasonable request (contact person: milenarovcanin@yahoo.com).
